# Poly-pharmacy among the elderly in a Danish emergency department

**DOI:** 10.1186/1757-7241-20-S2-P39

**Published:** 2012-04-16

**Authors:** Camilla Kobylecki, Thomas Andersen Schmidt

**Affiliations:** 1 The Emergency Department, Holbæk University Hospital, Denmark

## Background

A substantial part of the clientele in an emergency department setting are elderly patients who often consume several prescription medications daily hereby increasing the risk of potential drug-drug interactions. We examined the extent of poly-pharmacy at the time of admission among a group of elderly patients.

## Methods

All patients in the age range 65-79 years admitted consecutively to Holbaek Emergency Department during one week were included. Information on medication at the time of admission was obtained from medical charts.

## Results

A total of 73 patients, 48% women and 52% men were included, the average age being 72 years. The most frequent reasons for admission were lung diseases (21%), neurological disease (25%), cardiac diseases (15%) and infections (12%). The patients used on average 6.6 prescription medications daily, ranging from 0 to18 different drugs. 10 patients received 1 drug or less. The most frequently used drugs in this group were anti-hypertensive medication (56% of the patients), blood-cholesterol lowering drugs (55% of the patients), drugs to prevent thrombosis (56% of the patients), pain medication (37% of the patients) and diuretics (36% of the patients).

A total of 41 patients were prescribed anti-hypertensive medications. 20 patients were prescribed more than one drug to control blood pressure and 5 of these patients were treated with more than two different drugs to control blood pressure. 18 patients in anti-hypertensive treatment were also treated with diuretics. The most frequently prescribed anti-hypertensive drugs were calcium antagonists (n=15), beta-receptor blockers (n=15)and ACE-inhibitors (n=14).

A total of 40 patients were prescribed drugs to lower blood cholesterol and 75% (n=30) of these were treated with simvastatin. 41 patients were prescribed drugs to prevent thrombosis, among these acetylsalicylic acid (n=25), clopidogrel (n= 11) and warfarin (n= 10) were the most frequently used drugs.

27 patients were treated with pain medication at the time of admission and 15 of these patients received opioids.

## Conclusion

Cardiovascular drugs are the most predominantly prescribed drugs for elderly patients. Poly-pharmacy is in particular seen among antihypertensive medications. Caution in prescribing new drugs due to potential drug-drug interactions poses a challenge for the attending physician.

